# Supplementary material for: Dietary composition and feeding preference of Mantled guereza Colobus guereza (Rüppell, 1835), in Maze National Park, Ethiopia
Source: PeerJ. 2025 Feb 28;13:e18998. doi: 10.7717/peerj.18998 (PMC11874936; doi:10.7717/peerj.18998)
Supplement: Supplemental Information 1 — Table S1 Summary for monthly and seasonal feeding observation record for dry season (supplemental file) [file peerj-13-18998-s001.docx]

Table S1 Summary for monthly and seasonal feeding observation record for dry season (supplemental file)

| Month | Plant species | Frequency of records for plant parts consumed per plant species between seasons | | | | | | Total |
| --- | --- | --- | --- | --- | --- | --- | --- | --- |
|  |  | YL | ML | FR | Bk | Sh | FL |  |
| September | *Acacia polyacantha* | 0 | 0 | 0 | 13 | 0 | 0 | 13 |
| September | *Carissa spinarum* | 0 | 0 | 19 | 0 | 0 | 0 | 19 |
| September | *Grewia villosa* | 13 | 86 | 0 | 0 | 0 | 0 | 99 |
| September | *Millettia ferruginea* | 0 | 0 | 8 | 12 | 0 | 1 | 21 |
| September | *Moringa stenopetala* | 67 | 0 | 0 | 0 | 0 | 0 | 67 |
| September | *Syzygium guineense* | 1 | 66 | 0 | 0 | 0 | 32 | 99 |
| September | *Trichilia emetica* | 9 | 81 | 22 | 0 | 0 | 0 | 112 |
| September | Invertebrates | - | - | - | - | - | - | 8 |
| September | Sub-total | 90 | 233 | 49 | 25 | 0 | 33 | 438 |
| October | *Acacia polyacantha* | 0 | 0 | 0 | 10 | 0 | 0 | 10 |
| October | *Carissa spinarum* | 0 | 0 | 10 | 0 | 0 | 0 | 10 |
| October | *Grewia villosa* | 27 | 89 | 0 | 0 | 0 | 0 | 116 |
| October | *Millettia ferruginea* | 1 | 0 | 7 | 14 | 0 | 0 | 22 |
| October | *Moringa stenopetala* | 65 | 0 | 0 | 0 | 0 | 0 | 65 |
| October | *Syzygium guineense* | 0 | 66 | 0 | 0 | 3 | 36 | 105 |
| October | *Trichilia emetica* | 48 | 94 | 17 | 0 | 0 | 0 | 159 |
| October | Invertebrates | - | - | - | - | - | - | 5 |
| October | Sub-total | 141 | 249 | 34 | 24 | 3 | 36 | 492 |
| November | *Acacia polyacantha* | 0 | 0 | 0 | 13 | 0 | 0 | 13 |
| November | *Carissa spinarum* | 0 | 0 | 20 | 0 | 0 | 0 | 20 |
| November | *Grewia villosa* | 29 | 52 | 0 | 0 | 0 | 0 | 81 |
| November | *Millettia ferruginea* | 0 | 0 | 15 | 6 | 0 | 0 | 21 |
| November | *Moringa stenopetala* | 70 | 0 | 0 | 0 | 1 | 0 | 71 |
| November | *Syzygium guineense* | 18 | 45 | 29 | 0 | 0 | 30 | 122 |
| November | *Trichilia emetica* | 26 | 72 | 17 | 0 | 1 | 0 | 116 |
| November | Invertebrates | - | - | - | - | - | - | 8 |
| November | Sub-total | 143 | 169 | 81 | 19 | 2 | 30 | 452 |

Dry season data continued…

| Month | Plant species | Plant parts consumed | | | | | |  |
| --- | --- | --- | --- | --- | --- | --- | --- | --- |
|  |  | YL | ML | FR | Bk | Sh | FL |  |
| December | *Acacia polyacantha* | 0 | 0 | 0 | 14 | 0 | 0 | 14 |
| December | *Carissa spinarum* | 0 | 0 | 6 | 0 | 0 | 0 | 6 |
| December | *Grewia villosa* | 46 | 91 | 0 | 0 | 0 | 2 | 139 |
| December | *Ficus sycomorus* | 0 | 0 | 26 | 0 | 0 | 0 | 26 |
| December | *Millettia ferruginea* | 0 | 0 | 17 | 12 | 0 | 0 | 29 |
| December | *Moringa stenopetala* | 50 | 0 | 0 | 0 | 7 | 0 | 57 |
| December | *Syzygium guineense* | 3 | 27 | 21 | 0 | 0 | 19 | 70 |
| December | *Trichilia emetica* | 21 | 77 | 18 | 0 | 9 | 0 | 125 |
| December | Invertebrates | - | - | - | - | - | - | 9 |
| December | Sub-total | 120 | 195 | 88 | 26 | 16 | 21 | 475 |
| January | *Acacia polyacantha* | 0 | 0 | 0 | 17 | 0 | 0 | 17 |
| January | *Carissa spinarum* | 0 | 0 | 18 | 0 | 0 | 0 | 18 |
| January | *Grewia villosa* | 15 | 60 | 0 | 0 | 0 | 1 | 76 |
| January | *Millettia ferruginea* | 0 | 0 | 0 | 13 | 0 | 0 | 13 |
| January | *Moringa stenopetala* | 68 | 0 | 0 | 0 | 2 | 0 | 70 |
| January | *Syzygium guineense* | 20 | 41 | 23 | 0 | 0 | 39 | 123 |
| January | *Trichilia emetica* | 38 | 82 | 14 | 0 | 3 | 0 | 137 |
| January | Invertebrates | - | - | - | - | - | - | 9 |
| January | Sub-total | 141 | 183 | 55 | 30 | 5 | 40 | 463 |
| February | *Acacia polyacantha* | 0 | 0 | 0 | 16 | 0 | 0 | 16 |
| February | *Carissa spinarum* | 0 | 0 | 17 | 0 | 0 | 0 | 17 |
| February | *Grewia villosa* | 25 | 66 | 0 | 0 | 0 | 0 | 91 |
| February | *Ficus sycomorus* | 0 | 0 | 21 | 0 | 0 | 0 | 21 |
| February | *Millettia ferruginea* | 0 | 0 | 4 | 10 | 0 | 0 | 14 |
| February | *Moringa stenopetala* | 66 | 0 | 0 | 0 | 0 | 0 | 66 |
| February | *Syzygium guineense* | 11 | 44 | 19 | 0 | 0 | 25 | 99 |
| February | *Trichilia emetica* | 37 | 76 | 11 | 0 | 2 | 0 | 126 |
| February | Invertebrates | - | - | - | - | - | - | 16 |
| February | Sub-total | 139 | 186 | 72 | 26 | 2 | 25 | 466 |
| Total | | 774 | 1215 | 379 | 150 | 28 | 185 | 2786 |

**Note:** YL=young leaf, ML=mature leaf, FR=fruit, Bk=bark, Sh=shoot, FL=flower
