# Supplementary material for: Dietary composition and feeding preference of Mantled guereza Colobus guereza (Rüppell, 1835), in Maze National Park, Ethiopia
Source: PeerJ. 2025 Feb 28;13:e18998. doi: 10.7717/peerj.18998 (PMC11874936; doi:10.7717/peerj.18998)
Supplement: Supplemental Information 2 — Table S2 Summary for monthly and seasonal feeding observation record for wet season (supplemental file 2) [file peerj-13-18998-s002.docx]

Table S2 Summary for monthly and seasonal feeding observation record for wet season (supplemental file 2)

| Month | Plant species | Frequency of records for plant parts consumed per plant species between seasons | | | | | | Total |
| --- | --- | --- | --- | --- | --- | --- | --- | --- |
|  |  | YL | ML | FR | Bk | Sh | FL |  |
| March | *Acacia polyacantha* | 24 | 0 | 0 | 0 | 0 | 0 | 24 |
| March | *Carissa spinarum* | 0 | 0 | 27 | 0 | 0 | 0 | 27 |
| March | *Grewia villosa* | 68 | 18 | 0 | 0 | 0 | 0 | 86 |
| March | *Ficus sycomorus* | 0 | 0 | 0 | 0 | 0 | 0 | 0 |
| March | *Millettia ferruginea* | 0 | 0 | 0 | 0 | 0 | 0 | 0 |
| March | *Moringa stenopetala* | 0 | 0 | 0 | 0 | 0 | 0 | 0 |
| March | *Syzygium guineense* | 48 | 0 | 0 | 0 | 0 | 0 | 48 |
| March | *Trichilia emetica* | 171 | 22 | 0 | 0 | 31 | 0 | 224 |
| March | Invertebrates | - | - | - | - | - | - | 5 |
| March | *Sub-total* | 311 | 40 | 27 | 0 | 31 | 0 | 414 |
| April | *Acacia polyacantha* | 29 | 0 | 0 | 0 | 0 | 0 | 29 |
| April | *Carissa spinarum* | 0 | 0 | 30 | 0 | 0 | 0 | 30 |
| April | *Grewia villosa* | 59 | 13 | 0 | 0 | 0 | 0 | 72 |
| April | *Ficus sycomorus* | 0 | 0 | 0 | 0 | 0 | 0 | 0 |
| April | *Millettia ferruginea* | 0 | 0 | 0 | 0 | 0 | 0 | 0 |
| April | *Moringa stenopetala* | 0 | 0 | 0 | 0 | 0 | 0 | 0 |
| April | *Syzygium guineense* | 55 | 0 | 0 | 0 | 0 | 0 | 55 |
| April | *Trichilia emetica* | 143 | 13 | 0 | 0 | 30 | 0 | 186 |
| April | Invertebrates | - | - | - | - | - | - | 0 |
| April | *Sub-total* | 286 | 26 | 30 | 0 | 30 | 0 | 372 |
| May | *Acacia polyacantha* | 20 | 0 | 0 | 0 | 0 | 0 | 20 |
| May | *Carissa spinarum* | 0 | 0 | 36 | 0 | 0 | 0 | 36 |
| May | *Grewia villosa* | 59 | 23 | 0 | 0 | 0 | 0 | 82 |
| May | *Ficus sycomorus* | 0 | 0 | 0 | 0 | 0 | 0 | 0 |
| May | *Millettia ferruginea* | 0 | 0 | 0 | 0 | 0 | 0 | 0 |
| May | *Moringa stenopetala* | 9 | 0 | 0 | 0 | 0 | 0 | 9 |
| May | *Syzygium guineense* | 56 | 0 | 0 | 0 | 0 | 0 | 56 |
| May | *Trichilia emetica* | 152 | 24 | 0 | 0 | 38 | 0 | 214 |
| May | Invertebrates | - | - | - | - | - | - | 3 |
| May | *Sub-total* | 296 | 47 | 36 | 0 | 38 | 0 | 420 |

Wet season continued…

| Month | Plant species | Plant parts consumed | | | | | | Total |
| --- | --- | --- | --- | --- | --- | --- | --- | --- |
|  |  | YL | ML | FR | Bk | Sh | FL |  |
| June | *Acacia polyacantha* | 26 | 0 | 0 | 0 | 0 | 0 | 26 |
| June | *Carissa spinarum* | 0 | 0 | 24 | 0 | 0 | 0 | 24 |
| June | *Grewia villosa* | 62 | 21 | 0 | 0 | 0 | 0 | 83 |
| June | *Ficus sycomorus* | 0 | 0 | 0 | 0 | 0 | 0 | 0 |
| June | *Millettia ferruginea* | 0 | 0 | 0 | 0 | 0 | 0 | 0 |
| June | *Moringa stenopetala* | 0 | 0 | 0 | 0 | 0 | 0 | 0 |
| June | *Syzygium guineense* | 53 | 0 | 0 | 0 | 0 | 0 | 53 |
| June | *Trichilia emetica* | 161 | 15 | 0 | 0 | 35 | 0 | 211 |
| June | Invertebrates | - | - | - | - | - | - | - |
| June | Sub-total | 302 | 36 | 24 | 0 | 35 | 0 | 397 |
| July | *Acacia polyacantha* | 32 | 0 | 0 | 0 | 0 | 0 | 32 |
| July | *Carissa spinarum* | 0 | 0 | 27 | 0 | 0 | 0 | 27 |
| Jluy | *Grewia villosa* | 64 | 15 | 0 | 0 | 0 | 0 | 79 |
| July | *Ficus sycomorus* | 0 | 0 | 0 | 0 | 0 | 0 | 0 |
| July | *Millettia ferruginea* | 0 | 0 | 0 | 0 | 0 | 0 | 0 |
| July | *Moringa stenopetala* | 0 | 0 | 0 | 0 | 0 | 0 | 0 |
| July | *Syzygium guineense* | 49 | 0 | 0 | 0 | 0 | 0 | 49 |
| July | *Trichilia emetica* | 179 | 21 | 0 | 0 | 33 | 0 | 233 |
| July | Invertebrates | - | - | - | - | - | - | - |
| July | Sub-total | 324 | 36 | 27 | 0 | 33 | 0 | 420 |
| August | *Acacia polyacantha* | 27 | 0 | 0 | 0 | 0 | 0 | 27 |
| August | *Carissa spinarum* | 0 | 0 | 0 | 0 | 0 | 0 | 0 |
| August | *Grewia villosa* | 59 | 23 | 0 | 0 | 0 | 0 | 82 |
| August | *Ficus sycomorus* | 0 | 0 | 0 | 0 | 0 | 0 | 0 |
| August | *Millettia ferruginea* | 0 | 0 | 0 | 0 | 0 | 0 | 0 |
| August | *Moringa stenopetala* | 0 | 0 | 0 | 0 | 0 | 0 | 0 |
| August | *Syzygium guineense* | 47 | 0 | 0 | 0 | 0 | 0 | 47 |
| August | *Trichilia emetica* | 142 | 33 | 0 | 0 | 28 | 0 | 203 |
| August | Invertebrates | - | - | - | - | - | - | - |
| August | Sub-total | 275 | 56 | 0 | 0 | 28 | 0 | 359 |
|  | Total | 1794 | 241 | 144 | 0 | 195 | 0 | 2382 |
